# Elevated monocyte-to-high-density lipoprotein ratio is associated with increased risk of cognitive impairment and severe cerebral small vessel disease burden

**DOI:** 10.3389/fnagi.2025.1588488

**Published:** 2025-06-18

**Authors:** Yueshan Zhao, Meixi Li, Juan Zhang, Chao Wang, Mengyao Zhao, Qishuo Yang, Tianjun Wang, Peiyuan Lv

**Affiliations:** ^1^Department of Neurology, Hebei Medical University, Shijiazhuang, China; ^2^Department of Neurology, Hebei General Hospital, Shijiazhuang, China; ^3^Hebei Provincial Key Laboratory of Cerebral Networks and Cognitive Disorders, Shijiazhuang, China; ^4^Department of Rehabilitation, Hebei General Hospital, Shijiazhuang, China; ^5^Postdoctoral Innovation Base, Hebei General Hospital, Shijiazhuang, China; ^6^Department of Ethics Office, Hebei General Hospital, Shijiazhuang, China; ^7^Collaborative Innovation Center of Hebei Province for Mechanism, Diagnostics and Treatment of Neuropsychiatric Diseases, Shijiazhuang, China

**Keywords:** cerebral small vessel disease, total CSVD burden, monocyte count, cerebral ischemia, cognitive impairment, MHR

## Abstract

**Background:**

Monocyte-to-high-density lipoprotein ratio (MHR), as a novel biomarker, has shown potential in predicting the onset and progression of various diseases. However, the relationship between MHR and cerebral small vessel disease (CSVD) as well as cognitive impairment (CI), which are inflammation-related conditions remains unclear. This research explores the relationship between MHR and total CSVD burden as well as CI.

**Methods:**

This retrospective analysis included 212 eligible patients. On the basis of Mini-Mental State Examination (MMSE) scores, patients were classified into CI and no CI groups. Total CSVD burden was assessed using a composite score incorporating four MRI-based imaging markers. Participants were further stratified into mild and severe CSVD burden groups. MHR was determined by dividing the blood monocyte count by the high-density lipoprotein (HDL) concentration. Statistical analyses, including logistic regression, trend tests, restricted cubic spline modeling, and mediation analysis, were conducted using SPSS 26.0 and R software to explore the associations of MHR with CI, and CSVD burden.

**Results:**

Non-parametric analysis revealed that patients with CI and those with severe CSVD burden exhibited significantly higher MHR levels (*p* < 0.05) compared to their respective counterparts. Multivariable logistic regression identified elevated MHR (OR = 1.462, 95%CI: 1.057–2.022, *p* = 0.022) and severe CSVD burden (OR = 2.456, 95%CI: 1.306–4.617, *p* = 0.005) as significant risk factors for CI. Additionally, higher MHR levels were independently associated with severe CSVD burden (OR = 1.596, 95%CI: 1.092–2.334, *p* = 0.016). Compared to the lowest MHR tertile, the highest tertile exhibited a remarkably higher risk of CI (OR = 3.743, 95%CI: 1.557–8.995; *P*_trend_ = 0.010) and severe CSVD burden (OR = 2.594, 95%CI: 1.086–6.195; *P*_trend_ = 0.019). Restricted cubic spline analysis confirmed a non-linear association between MHR and both CI and severe CSVD burden. Mediation analysis further demonstrated that CSVD burden significantly mediated the relationship between MHR and CI.

**Conclusion:**

Elevated MHR is related to increased CSVD burden and CI. The mediating roles of severe CSVD burden indicates that a high MHR level may contribute to the progression of CSVD, thereby elevating the risk of CI.

## 1 Introduction

Cerebral small vessel disease (CSVD) involves multiple underlying pathological changes affecting the brain’s perforating arterioles, capillaries, and venules (Wardlaw et al., 2013a). It is a major contributor to stroke (Pantoni, 2010; Werring et al., 2025), cognitive impairment (CI), and dementia associated with aging (Hainsworth et al., 2024; Elahi et al., 2023). As populations age and life expectancy increases, CSVD has emerged as a leading vascular cause of cognitive decline, imposing a significant burden on healthcare systems worldwide (Jacob et al., 2023).

Magnetic resonance imaging (MRI) is an important method for assessing the occurrence, progression, and severity of CSVD. Typical MRI findings of CSVD include lacunes, total brain atrophy, white matter hyperintensity (WMH), enlarged perivascular space (EPVS), cerebral microbleeds (CMBs), and recent small subcortical infarcts (Wardlaw et al., 2013b). However, different CSVD markers correspond to distinct underlying pathophysiological mechanisms. CSVD is now recognized as a complex and dynamic condition (Ter Telgte et al., 2018; Shi and Wardlaw, 2016). To provide a comprehensive assessment of disease severity, Staals et al. introduced the total CSVD burden score, which integrates multiple MRI features of CSVD. This composite measure offers a more accurate representation of overall brain injury than any single imaging marker, making it a valuable tool for evaluating disease severity (Staals et al., 2014). Numerous studies have demonstrated a strong association between CSVD burden and CI (Elahi et al., 2023; Li et al., 2021).

Despite the significant burden of CSVD and its associated CI, no specific therapies currently exist due to an incomplete understanding of its underlying pathophysiological mechanisms. Therefore, early identification and intervention are crucial in preventing the onset and progression of cognitive decline. Recent research suggests that inflammation, a key pathological mechanism of CSVD and CI, plays a pivotal role in its development and progression (Zietz et al., 2024). Chronic inflammation has been closely linked to endothelial dysfunction, increased blood–brain barrier permeability, vascular oxidative stress, and impaired cerebral blood flow autoregulation, all of which may contribute to CSVD progression and cognitive decline (Hannawi, 2024). At the same time, CSVD itself can lead to several critical consequences that further exacerbate disease progression. These include endothelial dysfunction and impaired cerebral blood flow regulation, resulting in brain ischemia; increased microvascular fragility, leading to cerebral microhemorrhages; and disruption of the blood–brain barrier (BBB), which amplifies the inflammatory response (Ungvari et al., 2017; Nyúl-Tóth et al., 2024).

White blood cell subtypes, including neutrophils, lymphocytes, and monocytes, play a crucial role in all stages of inflammation (Hu et al., 2021). Conversely, high-density lipoproteins (HDL) possess strong anti-inflammatory and antioxidant properties (Bhale et al., 2024; Gkantzios et al., 2023). These contrasting characteristics have led to the emergence of novel inflammatory blood biomarkers, which have potential applications in routine clinical practice. Recent studies have demonstrated a significant association between inflammatory blood biomarkers, CSVD and CI, such as neutrophil-to-lymphocyte ratio, and systemic immune-inflammation index (Wang Y. et al., 2024; Hu et al., 2024; Cai et al., 2024; Xiao et al., 2023). These findings indicate the promising role of systemic inflammation in CSVD pathophysiology and suggest that blood-based biomarkers may serve as valuable tools for early detection and risk assessment. The monocyte-to-high-density lipoprotein ratio (MHR) has emerged as a promising prognostic marker, reflecting the interplay between inflammation and lipid metabolism in vascular disease pathophysiology (Pruc et al., 2024). Monocytes and HDL are widely available hematological markers that are cost-effective, easy to assess, and commonly used in clinical practice. As an inflammatory marker, MHR encapsulates both pro-inflammatory and anti-inflammatory mechanisms, with an elevated ratio indicating heightened systemic inflammation and diminished anti-inflammatory capacity. Previous research has revealed that MHR is associated with poor outcomes in cardiovascular (Sun et al., 2024; Jiang et al., 2022; Lin et al., 2024), malignant tumors (Miao et al., 2024) and atherosclerotic disease (Xi et al., 2022). As for cerebral disorders, MHR has also been shown to be closely related to acute ischemic stroke (Xu et al., 2023), hemorrhagic transformation post-stroke (Wang et al., 2020), psychiatric disorders (Villegas García et al., 2025) and migraine (Ulusoy, 2020). Additionally, emerging evidence suggests a correlation between MHR and imaging markers of CSVD (Nam et al., 2024). However, the relationships between total CSVD burden and MHR remain unclear, and research on its association with CI is still limited. This research aims to explores the relationships among MHR, total CSVD burden, and CI in hospitalized patients. In addition, mediation analysis was carried out to determine whether total CSVD burden mediates the effects of MHR on cognitive functions.

## 2 Materials and methods

### 2.1 Study design and participants

This retrospective analysis evaluated inpatient data from the Neurology Department of Hebei Provincial People’s Hospital between January 2022 and May 2024. Two hundred and twelve patients were consecutively enrolled based on predefined inclusion and exclusion criteria. All patient data were handled with strict confidentiality. The research was executed in compliance with the Declaration of Helsinki’s principles and granted ethical approval from the Ethical Committees of Hebei General Hospital (NO.2025-LW-0065).

#### 2.1.1 Inclusion criteria

(1) Age ≥50 years; (2) Completion of MRI scans using a 3.0 Tesla MR scanner, including T1-and T2-weighted imaging, T2 fluid-attenuated inversion recovery (FLAIR), and susceptibility-weighted imaging (SWI), which were necessary for evaluating CSVD markers; (3) availability of comprehensive blood biochemistry and complete blood count data; and (4) completion of cognitive function assessment.

#### 2.1.2 Exclusion criteria

(1) presence of acute stroke or severe neurological deficits; (2) History of major ischemic or hemorrhagic stroke, or other neurological disorders interfering with MRI-based CSVD assessment; (3) Presence of conditions linked to CI, such as traumatic brain injury, malignancy, epilepsy, thyroid dysfunction (hyperthyroidism or hypothyroidism), schizophrenia, carbon monoxide poisoning, depression, or anxiety; (4) Acute infections, malignancies, hematologic or autoimmune disorders, severe hepatic or renal dysfunction, or current use of immunosuppressants, glucocorticoids, or antibiotics that could influence inflammatory markers.

### 2.2 Demographic, clinical, and laboratory data collection

Patient demographic and clinical information were acquired from electronic medical records, including age, gender, education years, weight height, diastolic blood pressure (DBP), systolic blood pressure (SBP), smoking and alcohol consumption status, and medical history (cerebral infarction, hypertension, diabetes, and coronary heart disease). Body mass index (BMI) was determined by dividing weight (kg) by the square of height (m^2^).

Fasting venous blood specimens were collected within 24 h of hospital admission. Laboratory parameters recorded included fasting plasma glucose (FPG), total cholesterol (TC), triglycerides (TG), low-density lipoprotein (LDL), HDL, uric acid, serum total homocysteine, and complete blood counts (white blood cells, lymphocytes, neutrophils, monocytes, and other hematological indices). MHR was computed by dividing the blood monocyte count by the HDL concentration.

### 2.3 MRI acquisition and image analysis

All participants underwent brain MRI using a 3.0 Tesla scanner (Signa, GE Healthcare). The imaging protocol included T1-weighted imaging (T1WI), T2-weighted imaging (T2WI), fluid-attenuated inversion recovery (FLAIR), and susceptibility-weighted imaging (SWI). The specific scanning parameters were as follows: T1WI (TR/TE: 1909/20.2 ms, slice thickness: 5 mm); T2WI (TR/TE: 5000/125 ms, slice thickness: 5 mm); SWI (TR/TE: 78.6/47.6 ms, slice thickness: 2 mm); and FLAIR (TR/TE: 8502/159.4 ms, slice thickness: 5 mm).

The total CSVD burden was assessed by integrating four key MRI markers: WMH, CMBs, EPVS, and lacunes in the basal ganglia (Huijts et al., 2013). Two experienced neurologists, blinded to clinical data, independently assessed the images in compliance with the Standards for Reporting Vascular Changes on Neuroimaging guidelines (Wardlaw et al., 2013b). Any discrepancies were resolved by a senior radiologist. The CSVD burden scoring criteria included: (1) WMH: Defined as bilateral, predominantly symmetric hyperintensities on T2WI. Deep/periventricular WMH were graded using the Fazekas scale on T2WI and FLAIR sequences (Fazekas et al., 1993). A Fazekas score of 2–3 for deep WMH or 3 for periventricular WMH contributed 1 point to the total CSVD burden score. (2) CMBs: Identified as hypointense lesions (<10 mm) with clear margins on SWI. Deep CMBs (thalamus, basal ganglia, internal and external capsules, deep/periventricular WMH and corpus callosum) were assessed using the Microbleed Anatomical Rating Scale (Gregoire et al., 2009). Occurrence of deep CMBs contributed 1 point to the total score. (3) Lacunes: Defined as ovoid or round subcortical lesions (3–15 mm) with cerebrospinal fluid-like signal characteristics (hypointense on T1WI, hyperintense on T2WI) (Wardlaw et al., 2013b). Occurrence of ≥1 lacunes contributed 1 point. (4) EPVS: Small (<3 mm), round or linear cerebrospinal fluid-filled spaces adjacent to blood vessels, visible on T2WI without hyperintensity on FLAIR. EPVS in the basal ganglia were quantified using a visual grading scale: 0 (absent), 1 (1–10), 2 (11–20), 3 (21–40), 4 (>40) (Wardlaw et al., 2013b; Doubal et al., 2010). A basal ganglia EPVS score of 2 or higher contributed 1 point. The total CSVD burden score was computed as the sum of these 4 biomarkers. Patients were assigned to groups on the basis of their scores: mild CSVD burden (score 1–2) and severe CSVD burden (score 3–4) (Kim et al., 2021).

### 2.4 Cognitive function assessment

Cognitive functions were evaluated using the standardized Chinese version of the Mini-Mental State Examination (MMSE). Given the varying educational backgrounds of participants, education-adjusted cut-off scores were applied for CI diagnosis. The thresholds were as follows: illiterate: ≤17, primary education (1–6 years): ≤20, and higher education (>7 years): ≤24 (Li et al., 2016).

### 2.5 Statistical analysis

Data were assessed using SPSS v26.0. Descriptive statistics were expressed as mean ± standard deviation or median (Q25, Q75) for continuous data, and as counts with percentages for categorical data. Group comparisons were conducted using the t-test, χ^2^ test, or Mann–Whitney U test, as appropriate. Statistical significance was set at *p* < 0.05. Binary logistic regression analysis was executed to determine significant risk factors for CI and severe CSVD burden. Prior to multivariable regression, collinearity diagnostics were conducted to ensure that the selected variables were not highly correlated. No collinearity issues were detected in this study. To examine a potential dose–response relationship between MHR levels and CI or severe CSVD burden, participants were stratified into four quartile-based MHR groups. Each group was assigned its median value, and a trend test was conducted to assess the risk gradient for CI or severe CSVD burden. Restricted cubic spline analysis was conducted using the rms packages in R (version 4.4.2) to visualize potential linear or non-linear associations between MHR levels and CI or severe CSVD burden. This method also helped identify possible cutoff values. Restricted cubic spline analyses were performed using four knots, with *p* < 0.05 deemed statistically significant. Mediation analysis was conducted using R (version 4.4.2) with the mediation package. The analysis aimed to determine whether CSVD burden severity mediated the relationship between MHR levels and CI. To enhance the robustness of effect estimation, 5,000 bootstrap samples were used in each mediation analysis.

## 3 Result

### 3.1 Baseline features

Two hundred and twelve eligible patients were recruited. The median age was 67 years (IQR: 58–73), while the mean age was 66.23 ± 9.7 years. Among them, 128 (60.4%) were male. Common vascular risk factors included hypertension (61.3%), diabetes (30.2%), current smoking (21.7%), and previous stroke (31.6%), while fewer patients had other risk factors such as alcohol consumption (17%) and coronary heart disease (14.6%). See Table 1 for details.

**Table 1:** Demographic and clinical features of all participants based on cognitive status.

Variable	Total (*n* = 212)	No CI group (*n* = 113)	CI group (*n* = 99)	*p*-value
Age, median (IQR), year	67.0 (58–73)	65.0 (56–71)	69.0 (63–77)	< 0.001^*^
Sex (male), *n* (%)	128 (60.4)	65 (57.5)	63 (63.6)	0.364
Education, median (IQR), year	9 (6–12)	9 (9–12)	9 (6–12)	0.022^*^
BMI, median (IQR), kg/m^2^	24.79 (23.12–26.49)	25.00 (23.34–26.79)	24.34 (23.00–26.30)	0.151
Current smoking, *n* (%)	46 (21.7)	27 (23.9)	19 (19.2)	0.407
Alcohol use, *n* (%)	36 (17.0)	22 (19.5)	14 (14.1)	0.303
Hypertension, *n* (%)	130 (61.3)	66 (58.4)	64 (64.6)	0.352
SBP, median (IQR), mmHg	138 (125–149)	136 (121–147)	140 (129–154)	0.041^*^
DBP, median (IQR), mmHg	81 ± 12.1	80 ± 12.9	82 ± 11.1	0.241
Diabetes	64 (30.2)	25 (22.1)	39 (39.4)	0.006^*^
Coronary heart disease, *n* (%)	31 (14.6)	15 (13.3)	16 (16.2)	0.553
History of stroke	67 (31.6)	30 (26.5)	37 (37.4)	0.091
Monocyte counts	0.34 (0.27–0.42)	0.35 (0.25–0.43)	0.34 (0.28–0.42)	0.632
TC, median (IQR), mmol/L	4.20 ± 1.09	4.27 ± 1.05	4.11 ± 0.12	0.288
TG, median (IQR), mmol/L	1.11 (0.78–1.45)	1.15 (0.81–1.46)	1.02 (0.77–1.43)	0.466
HDL, median (IQR), mmol/L	1.15 (0.97–1.36)	1.15 (0.98–1.40)	1.14 (0.93–1.30)	0.241
LDL, median (IQR), mmol/L	2.56 ± 0.79	2.66 ± 0.80	2.51 ± 0.79	0.176
Uric acid, median (IQR), umol/L	292.00 (235.22–355.15)	306.40 (247.80–360.55)	281.58 (228.70–338.90)	0.087
Serum tHcy, median (IQR), umol/L	13.15 (10.00–17.35)	12.90 (10.00–15.60)	13.9 (10.00–19.00)	0.106
FPG, median (IQR), mmol/L	5.35 (4.84–6.31)	5.23 (4.71–5.86)	5.49 (4.93–6.59)	0.018^*^
MHR, median (IQR)	0.302 (0.232–0.414)	0.296 (0.210–0.386)	0.316 (0.252–0.458)	0.004^*^
Total CSVD burden score				< 0.001^*^
0, *n* (%)		31 (27.4)	8 (8.1)	
1, *n* (%)		19 (16.8)	13 (13.1)	
2, *n* (%)		29 (25.7)	15 (15.2)	
3, *n* (%)		16 (14.2)	26 (26.2)	
4, *n* (%)		18 (15.9)	37 (37.4)	

*p < 0.05. CI, cognitive impairment; SD, standard deviation; IQR, interquartile range; BMI, Body mass index; SBP, systolic blood pressure; DBP, diastolic blood pressure; FPG, fasting plasma glucose; TC, total cholesterol; TG, triglyceride; HDL, high density lipoprotein; LDL, low density lipoprotein; tHcy, total homocysteine; MHR, monocyte to high-density lipoprotein cholesterol ratio; CSVD, cerebral small vessel disease.

On the basis of MMSE scores, patients were categorized into two groups: those with CI and those without. The baseline features of both groups are displayed in Table 1. Ninety-nine (46.7%) patients were classified as having CI. Compared to patients without CI, those with CI were significantly older (63.8 ± 9.2 vs. 69.0 ± 9.8 years, *p* < 0.001), had lower education levels (*p* = 0.022), and exhibited a higher prevalence of diabetes (*p* = 0.006), elevated FPG (*p* = 0.018), and higher admission SBP (*p* = 0.041). Furthermore, the CI group demonstrated markedly higher MHR levels and total CSVD burden scores than the no-CI group (*p* = 0.004, *p* < 0.001). See Table 1 for details.

Patients were also stratified into 2 groups on the basis of total CSVD burden scores: the mild CSVD burden group (≤2 points) and the severe CSVD burden group (>2 points). A total of 97 (45.8%) patients were assigned to the severe CSVD burden group. The characteristics of these 2 groups are displayed in Table 2. Patients in the severe CSVD burden group were significantly older (*p* < 0.001), demonstrated a greater proportion of males (*p* = 0.017), received fewer years of education (*p* = 0.006), and exhibited a greater prevalence of hypertension (*p* < 0.001), diabetes (*p* = 0.044), and previous stroke (*p* < 0.001). Additionally, these patients exhibited higher SBP (*p* = 0.001), DBP (*p* = 0.006), TC (*p* = 0.024), and serum total homocysteine levels (*p* = 0.024), along with increased MHR levels (*p* = 0.001) but lower HDL levels (*p* = 0.002) and MMSE scores (*p* < 0.001). See Table 2 for further details.

**Table 2:** Demographic and clinical features of all participants based on CSVD burden severity.

Variable	Total (*n* = 212)	Mild CSVD burden group (*n* = 115)	Severe CSVD burden group (*n* = 97)	*p*-value
Age, median (IQR), year	67.0 (58–73)	65.0 (55–72)	70.0 (64–75)	< 0.001^*^
Sex (male), *n* (%)	128 (60.4)	61 (53.0)	67 (69.1)	0.017^*^
Education, median (IQR), year	9 (6–12)	9 (9–12)	9 (6–12)	0.006^*^
BMI, median (IQR), kg/m^2^	24.79 (23.12–26.49)	24.98 (22.86–26.49)	24.44 (23.14–26.56)	0.884
Current smoking, *n* (%)	46 (21.7)	22 (19.1)	24 (24.7)	0.323
Alcohol use, *n* (%)	36 (17.0)	15 (13.0)	21 (21.6)	0.096
Hypertension, *n* (%)	130 (61.3)	55 (47.8)	75 (77.3)	< 0.001^*^
SBP, median (IQR), mmHg	138 (125–149)	135 (121–146)	140 (130–159)	0.001^*^
DBP, median (IQR), mmHg	81 ± 12.1	79 ± 11.7	84 ± 12.2	0.006^*^
Diabetes	64 (30.2)	28 (24.3)	36 (37.1)	0.044^*^
Coronary heart disease, *n* (%)	31 (14.6)	16 (13.9)	15 (15.5)	0.750
History of stroke	67 (31.6)	25 (21.7)	42 (43.3)	0.001^*^
Monocyte counts	0.34 (0.27–0.42)	0.33 (0.26–0.42)	0.35 (0.28–0.43)	0.297
TC, median (IQR), mmol/L	4.20 ± 1.09	4.35 ± 1.09	4.01 ± 1.07	0.024^*^
TG, median (IQR), mmol/L	1.11 (0.78–1.45)	1.08 (0.77–1.42)	1.14 (0.82–1.52)	0.358
HDL, median (IQR), mmol/L	1.15 (0.97–1.36)	1.18 (1.05–1.44)	1.08 (0.91–1.29)	0.002^*^
LDL, median (IQR), mmol/L	2.56 ± 0.79	2.67 ± 0.83	2.48 ± 0.75	0.066
Uric acid, median (IQR), umol/L	292.00 (235.20–355.20)	292.60 (233.50–350.10)	290.80 (238.40–363.05)	0.524
Serum tHcy, median (IQR), umol/L	13.20 (10.00–17.40)	12.40 (9.80–16.00)	14.2 (10.60–18.35)	0.024^*^
FPG, median (IQR), mmol/L	5.35 (4.84–6.31)	5.25 (4.85–6.00)	5.47 (4.80–6.67)	0.147
MHR, median (IQR)	0.302 (0.232–0.414)	0.296 (0.223–0.377)	0.352 (0.247–0.469)	0.001^*^
MMSE score	24 (19–27)	26 (23–28)	21 (16–24)	< 0.001^*^

*p < 0.05. SD, standard deviation; IQR, interquartile range; BMI, Body mass index; SBP, systolic blood pressure; DBP, diastolic blood pressure; FPG, fasting plasma glucose; TC, total cholesterol; TG, triglyceride; HDL, high density lipoprotein; LDL, low density lipoprotein; tHcy, total homocysteine; MHR, monocyte to high-density lipoprotein cholesterol ratio; CSVD, cerebral small vessel disease; MMSE, Mini-Mental State Examination.

### 3.2 MHR and CI

A binary logistic regression model was employed to examine the relationship between MHR and CI. Initially, univariate logistic regression analysis identified several significant risk factors for CI, including age (OR = 1.060, 95%CI: 1.029–1.092; *p* < 0.001), diabetes (OR = 2.288, 95%CI: 1.256–4.168; *p* = 0.007), FPG (OR = 1.261, 95%CI: 1.048–1.517; *p* = 0.014), severe CSVD burden (OR = 4.066, 95%CI: 2.291–7.218; *p* < 0.001), and MHR (OR = 1.632, 95%CI: 1.222–2.180; *p* = 0.001). Conversely, higher education level was a protective factor against CI (OR = 0.901, 95%CI: 0.835–0.972; *p* = 0.007). After adjusting for confounders such as age, education, diabetes, FPG, and severe CSVD burden, multivariable logistic regression confirmed MHR as a significant risk factor for CI (OR = 1.462, 95%CI: 1.057–2.022; *p* = 0.022) (Table 3).

**Table 3:** Logistic regression modeling of potential predictors for CI.

Variable	Univariable analysis		Multivariable analysis	
	OR (95% CI)	*p*-value	OR (95% CI)	*p*-value
Age	1.060 (1.029–1.092)	< 0.001^*^	1.043 (1.009–1.079)	0.014^*^
Sex	1.292 (0.743–2.249)	0.364		
Education	0.901 (0.835–0.972)	0.007^*^	0.930 (0.854–1.011)	0.089
Body mass index	0.934 (0.866–1.007)	0.075		
Current smoking	0.756 (0.391–1.465)	0.408		
Alcohol use	0.681 (0.328–1.417)	0.304		
Hypertension	1.302 (0.746–2.272)	0.352		
SBP	1.013 (0.998–1.027)	0.084		
DBP	1.014 (0.991–1.037)	0.240		
Diabetes	2.288 (1.256–4.168)	0.007^*^	1.466 (0.705–3.047)	0.306
Coronary heart disease	1.259 (0.587–2.700)	0.553		
History of stroke	1.651 (0.921–2.958)	0.092		
Monocyte counts	0.701 (0.256–1.923)	0.491		
TC	0.872 (0.679–1.122)	0.287		
TG	1.189 (0.805–1.756)	0.385		
HDL	0.571 (0.229–1.424)	0.229		
LDL	0.789 (0.559–1.113)	0.177		
Uric acid	1.000 (0.998–1.002)	0.910		
Serum tHcy	1.032 (0.990–1.076)	0.140		
FPG	1.261 (1.048–1.517)	0.014^*^	1.127 (0.908–1.399)	0.278
Severe CSVD burden	4.066 (2.291–7.218)	< 0.001^*^	2.456 (1.306–4.617)	0.005^*^
MHR	1.632 (1.222–2.180)	0.001^*^	1.462 (1.057–2.022)	0.022^*^

*p < 0.05. CI, cognitive impairment; SBP, systolic blood pressure; DBP, diastolic blood pressure; FPG, fasting plasma glucose; TC, total cholesterol; TG, triglyceride; HDL, high density lipoprotein; LDL, low density lipoprotein; tHcy, total homocysteine; MHR, monocyte to high-density lipoprotein cholesterol ratio; CSVD, cerebral small vessel disease.

To further investigate the association between MHR levels and CI, a trend analysis was performed. Patients were stratified into 4 groups according to MHR quartiles: Tertile 1 (≤0.232), Tertile 2 (0.232–0.302), Tertile 3 (0.302–0.414), and Tertile 4 (≥0.414). A significant trend was observed, with higher MHR levels correlating with a higher risk of CI (OR = 3.667, 95%CI: 1.622–8.289; *P*_trend_ = 0.007). This association remained significant after adjustment for age, gender, and education (OR = 3.743, 95%CI: 1.557–8.995; *P*_trend_ = 0.010) (Table 4).

**Table 4:** ORs (and 95% CIs) of CI and severe CSVD burden score according to tertiles of MHR levels.

	MHR levels, median (range)				
	Quartile 1 (≤0.232)	Quartile 2 (0.232–0.302)	Quartile 3 (0.302–0.414)	Quartile 4 (≥0.414)	*p*-value for trend^a^
Cognitive impairment					
Model 1	1.00 (reference)	2.513 (1.122–5.630)	1.731 (0.770–3.893)	3.667 (1.622–8.289)	0.007
Model 2	1.00 (reference)	2.513 (1.068–5.915)	1.817 (0.767–4.304)	3.743 (1.557–8.995)	0.010
Severe CSVD burden score					
Model 1	1.00 (reference)	1.122 (0.510–2.466)	1.038 (0.471–2.290)	3.006 (1.352–6.681)	0.005
Model 2	1.00 (reference)	0.920 (0.388–2.181)	0.910 (0.382–2.167)	2.594 (1.086–6.195)	0.019

a Tests for trend were conducted by treating the tertiles as a continuous variable and assigning the median for each quintile.

Model 1: Unadjusted; Model 2: Adjusted for age, sex and education.

**Figure 1:**
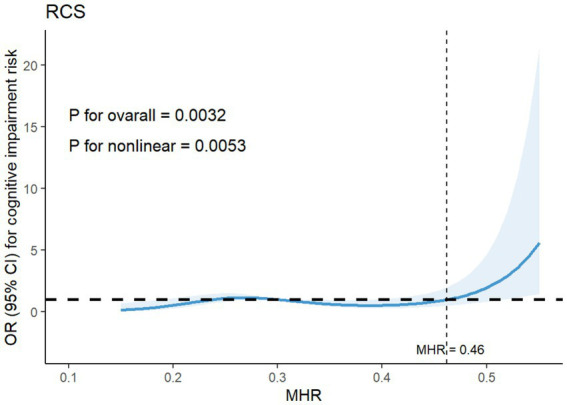
Restricted cubic spline for the association between MHR levels and the risk of cognitive impairment. Adjusted for age, diabetes, total CSVD burden, education, FPG. MHR, monocyte to high-density lipoprotein cholesterol ratio; CSVD, cerebral small vessel disease; FPG, fasting plasma glucose.

A restricted cubic spline analysis was conducted to provide a detailed visualization of the dose–response relationships between MHR levels and CI risk. The analysis revealed a non-linear association (*P*_non-linearity_ = 0.0053), indicating that CI risk remained relatively low at lower MHR levels. However, when MHR exceeded cutoff value of 0.46, there was a marked increase in the likelihood of CI (Figure 1).

### 3.3 MHR and CSVD burden

In an unadjusted binary logistic regression analysis, higher MHR levels were markedly linked to a higher risk of severe CSVD burden (OR = 1.760, 95%CI: 1.309–2.365; *p* < 0.001). Additionally, severe CSVD burden demonstrated significant correlations with several factors, including age (OR = 1.066, 95%CI: 1.033–1.099; *p* < 0.001), sex (OR = 1.977, 95%CI: 1.123–3.479; *p* = 0.018), education level (OR = 0.885, 95%CI: 0.819–0.956; *p* = 0.002), hypertension (OR = 3.719, 95%CI: 2.042–6.775; *p* < 0.001), SBP (OR = 1.029, 95%CI: 1.013–1.045; *p* < 0.001), DBP (OR = 1.033, 95%CI: 1.009–1.057; *p* = 0.007), diabetes (OR = 1.834, 95%CI: 1.014–3.316; *p* = 0.045), prior stroke history (OR = 2.749, 95%CI: 1.512–4.999; *p* = 0.001), TC (OR = 0.744, 95%CI: 0.574–0.965; *p* = 0.026), HDL (OR: 0.203, 95%CI: 0.047–0.552; p = 0.002), and serum tHcy (OR = 1.044, 95%CI: 1.000–1.089; *p* = 0.048). After adjusting for potential confounders in a multivariable binary logistic regression model, MHR remained a significant predictor of severe CSVD burden (OR = 1.596, 95%CI: 1.092–2.334; *p* = 0.016) (Table 5).

**Table 5:** Logistic regression modeling of potential predictors for severe CSVD burden.

Variable	Univariable analysis		Multivariable analysis	
	OR (95% CI)	*p*-value	OR (95% CI)	*p*-value
Age	1.066 (1.033–1.099)	< 0.001^*^	1.083 (1.041–1.127)	< 0.001^*^
Sex	1.977 (1.123–3.479)	0.018^*^	2.226 (1.025–4.839)	0.043^*^
Education	0.885 (0.819–0.956)	0.002^*^	0.854 (0.772–0.945)	0.002^*^
Body mass index	0.980 (0.921–1.043)	0.526		
Current smoking	1.390 (0.722–2.675)	0.324		
Alcohol use	1.842 (0.891–3.810)	0.099		
Hypertension	3.719 (2.042–6.775)	< 0.001^*^	3.241 (1.516–6.928)	0.002^*^
SBP	1.029 (1.013–1.045)	< 0.001^*^	1.010 (0.988–1.032)	0.395
DBP	1.033 (1.009–1.057)	0.007^*^	1.042 (1.004–1.080)	0.028^*^
Diabetes	1.834 (1.014–3.316)	0.045^*^	1.397 (0.655–2.979)	0.387
Coronary heart disease	1.132 (0.528–2.427)	0.750		
History of stroke	2.749 (1.512–4.999)	0.001^*^	1.817 (0.854–3.864)	0.121
Monocyte counts	1.977 (0.434–9.001)	0.378		
TC	0.744 (0.574–0.965)	0.026^*^	0.950 (0.676–1.335)	0.767
TG	1.376 (0.918–2.063)	0.122		
HDL	0.203 (0.047–0.552)	0.002^*^		
LDL	0.722 (0.502–1.024)	0.067		
Uric acid	1.000 (0.998–1.001)	0.664		
Serum tHcy	1.044 (1.000–1.089)	0.048^*^	1.023 (0.969–1.081)	0.414
FPG	1.170 (0.983–1.393)	0.078		
MHR	1.760 (1.309–2.365)	< 0.001^*^	1.596 (1.092–2.334)	0.016^*^

*p < 0.05. SBP, systolic blood pressure; DBP, diastolic blood pressure; FPG, fasting plasma glucose; TC, total cholesterol; TG, triglyceride; HDL, high density lipoprotein; LDL, low density lipoprotein; tHcy, total homocysteine; MHR, monocyte to high-density lipoprotein cholesterol ratio; CSVD, cerebral small vessel disease.

A significant trend was found between MHR tertiles and the risk of severe CSVD burden. Specifically, patients in the highest MHR tertile (≥0.414) had a markedly greater likelihood of severe CSVD burden compared to those in the lowest tertile (≤0.232) (OR = 3.006, 95%CI: 1.352–6.681; *P*_trend_ = 0.005). This relationship continued to be statistically significant, though slightly attenuated, following adjustment for age, gender, and education (OR = 2.594, 95%CI: 1.086–6.195; *P*_trend_ = 0.019) (Table 4).

**Figure 2:**
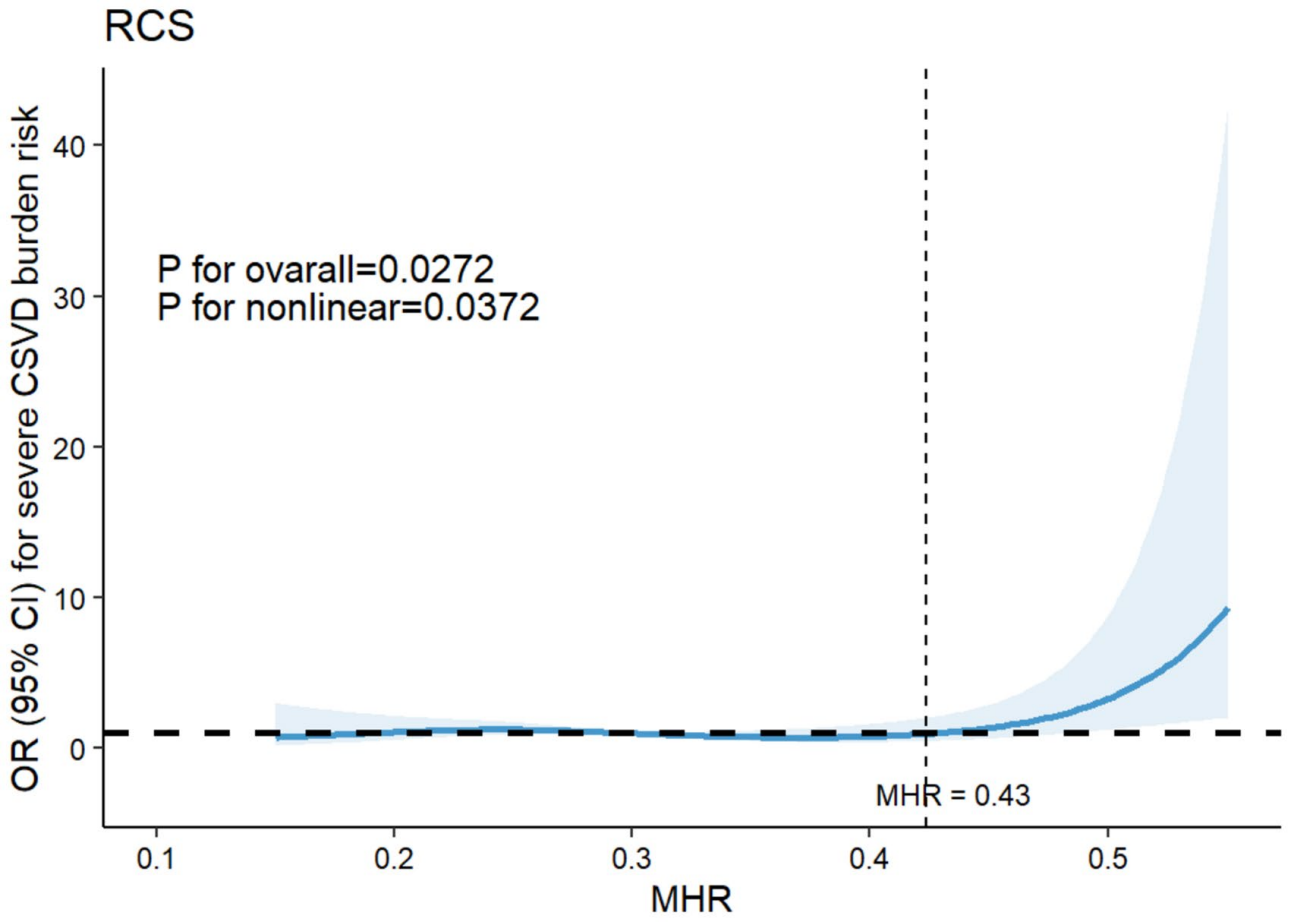
Restricted cubic spline for the association between serum MHR levels and the risk of severe CSVD burden. Adjusted for age, sex, diabetes, hypertension, SBP, DBP, education, stroke, serum tHcy, TC. MHR, monocyte to high-density lipoprotein cholesterol ratio; CSVD, cerebral small vessel disease; SBP, systolic blood pressure; DBP, diastolic blood pressure; tHcy, total homocysteine; TC, total cholesterol.

To further illustrate the dose–response relationships between MHR levels and severe CSVD burden, a restricted cubic spline analysis was performed. The graphical representation indicated a non-linear correlation (*P*_non-linearity_ = 0.0372), with the risk of severe CSVD burden remaining relatively stable at lower MHR levels but rising sharply once MHR exceeded approximately the cutoff of 0.43 (Figure 2).

### 3.4 Mediation effect of severe CSVD burden

**Figure 3:**
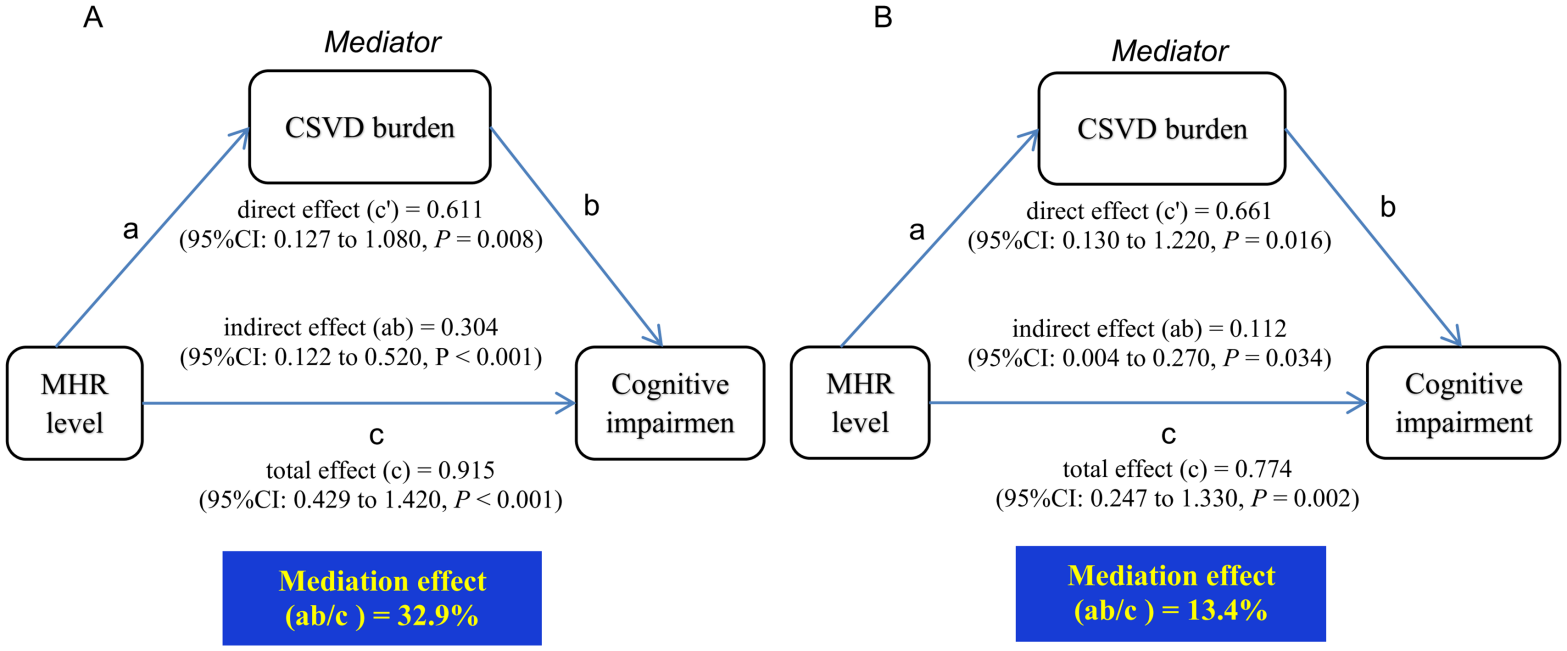
Mediation analysis of severe CSVD burden in the relationships between MHR levels and CI. **(A)** Unadjusted for potential confounders. **(B)** Adjusted for age, diabetes, education, FPG, sex, hypertension, SBP, DBP, history of stroke, TC, serum tHcy. CI, cognitive impairment; FPG, fasting plasma glucose; SBP, systolic blood pressure; DBP, diastolic blood pressure; TC, total cholesterol; tHcy, total homocysteine; MHR, monocyte to high-density lipoprotein cholesterol ratio; CSVD, cerebral small vessel disease.

The previous analyses revealed that higher MHR levels were related to a higher risk of both severe CSVD burden and CI, and CSVD also identified as a significant risk factor for CI. This section of the study employed a mediation model to assess whether severe CSVD burden acts as a mediator in the relationships between MHR levels and CI. The mediation analysis results are presented in Figure 3.

MHR levels exhibited a direct effect on CI (c’ = 0.611, 95%CI: 0.127–1.080; *p* = 0.008) as well as a significant total effect (c = 0.915, 95%CI: 0.429–1.420; *p* < 0.001). Incorporating severe CSVD burden into the model revealed a significant indirect effect (ab = 0.304, 95%CI: 0.122–0.520; *p* < 0.001), implying that severe CSVD accounted for 32.9% of the overall impact of MHR on CI (Figure 3A).

After adjustment for potential confounding factors, the mediation effect of severe CSVD burden remained significant (ab = 0.112, 95% CI: 0.004–0.270; *p* = 0.034), with 13.4% of the overall impact of MHR on CI being attributable to severe CSVD burden (Figure 3B).

## 4 Discussion

This retrospective study assessed the associations of MHR levels with CI and severe CSVD burden. Our findings highlighted an obvious association between MHR and CI, with individuals in the CI group exhibiting higher MHR levels compared to those in no CI group. Additionally, elevated MHR emerged as a significant risk factor for CI. Specifically, as MHR increased, the risk of CI also rose. Interestingly, the relationship between MHR and CI was not strictly linear. Restricted cubic spline analysis confirmed a nonlinear dose–response association, with a dose–response relationship observed obviously within a relatively higher range of MHR levels. A notable increase in the risk of CI was observed when MHR exceeded 0.46. A similar trend was identified between MHR and total CSVD burden, where higher MHR levels represented a significant risk factor. The risk of severe CSVD burden also followed a nonlinear pattern, significantly increasing when MHR surpassed 0.43. Furthermore, our study established a strong correlation between total CSVD burden and CI. Mediation analysis demonstrated that severe CSVD burden partially mediated the relationship between MHR and CI, suggesting that elevated MHR may contribute to worsening CSVD burden and subsequently increases the likelihood of cognitive decline. These findings reinforce the potential role of MHR as a biomarker for both vascular brain injury and cognitive dysfunction.

Regarding the relationship between the MHR and CSVD, a recent study has reported a strong association between MHR and CSVD imaging markers, including WMH, lacunar infarctions, and microbleeds. Additionally, MHR emerged as a significant risk factor for these features, further elucidating the dose–response relationships between MHR levels and CSVD characteristics. Evidence suggests that MHR is a more reliable indicator of the inflammatory state in CSVD than NLR, given that CSVD is driven by chronic inflammation, with monocytes playing a crucial role in this process (Nam et al., 2024). A study investigating MHR and migraines also observed a positive correlation between MHR levels and WMH (Ulusoy, 2020). However, we did not analyze individual imaging features separately, as multiple studies indicate that total CSVD burden is a more comprehensive measure of cerebral small vessel disease severity. Our results confirm that MHR is a significant risk factor for severe CSVD burden, and there is a dose–response relationship between the two. Trend analysis revealed a slight decrease in the OR value for the middle tertile, suggesting that the association between MHR and total CSVD burden may not strictly linear. Further, restricted cubic spline analysis confirmed a non-linear relationship, showing that the risk of severe CSVD burden significantly rises when MHR exceeds 0.43.

As for the relationship between MHR and cognitive impairment, a study on Parkinson’s disease (PD) and Parkinsonism revealed that MHR is associated with both CI and functional disability in PD patients. Specifically, in the PD group, higher MHR levels were negatively correlated with MMSE scores (Kwak et al., 2024). This finding aligns with our results, suggesting that elevated MHR increases the risk of CI, particularly when MHR surpasses 0.46. As highlighted earlier, inflammation plays a crucial role in cognitive decline, with monocytes serving as key indicators of chronic inflammation associated with neurodegeneration. Additionally, substantial research has shown that HDL cholesterol plays a protective role in cognitive function by enhancing endothelial nitric oxide synthase (eNOS) activity, reducing neuroinflammation, inhibiting vascular adhesion, and clearing excess cholesterol from the brain (Avula et al., 2022). Growing evidence supports the notion that higher HDL levels and its primary apolipoprotein A-I are related to a lower risk of dementia in the elderly population (Norton et al., 2014; Wang R. et al., 2024). The Baltimore Longitudinal Study of Aging found that individuals with higher baseline HDL cholesterol levels had a lower likelihood of CI decades later. Similarly, the InChianti study demonstrated that patients with dementia had significantly lower HDL cholesterol levels (Michikawa, 2003). The established relationship between monocytes, HDL, and CI further reinforces our findings, confirming the strong association between MHR and cognitive decline.

Although research specifically examining MHR and vascular cognitive impairment (VCI) remains limited, existing evidence suggests a strong relationship between MHR and its associated risk factors. Firstly, MHR has been closely related to both the onset and prognosis of stroke. Elevated MHR could serve as an independent predictor of all-cause mortality and worsen functional outcomes in transient ischemic attack or ischemic stroke patients (Xu et al., 2023). Additionally, other studies have explored MHR as a potential blood biomarker for predicting stroke progression (Bi et al., 2021; Bolayir et al., 2018). Secondly, research into MHR and hypertension has demonstrated a strong correlation between MHR and hypertension risk, classification, and associated organ damage (Toprak et al., 2024; Kaplan et al., 2020; Meng X. et al., 2024). Thirdly, the relationship between MHR and diabetes, as well as its complications, has been extensively studied. A prospective study tracking 40,813 non-diabetic participants found that elevated MHR levels were markedly related to a higher risk of developing diabetes, as determined by multivariable Cox regression analysis (Wu et al., 2024). A separate cross-sectional study in China revealed a positive correlation between MHR and prediabetes prevalence, with a non-linear relationship observed (Ruan et al., 2024). Additionally, research on diabetic nephropathy suggests that MHR could serve as a marker for both the presence and progression of diabetic kidney disease (Yang et al., 2025). These findings collectively demonstrate that higher MHR is linked to an elevated risk of CI-related risk factors. Given that CSVD and VCI share multiple common risk factors, this evidence further supports the observed relationships between MHR, CI, and CSVD burden in our study.

From the perspective of pathogenesis, inflammation not only attracts attention in large artery diseases but also garners significant concern in small vessel diseases. Research has demonstrated that both systemic and central nervous system inflammation are closely associated with CSVD (Walsh et al., 2021; Nyúl-Tóth et al., 2024). Moreover, inflammation is an essential driver in the onset and progression of CI (Tack et al., 2023; Pan and Ma, 2024; Xiao et al., 2023). Systemic inflammation impacts CSVD and cognitive function through multiple pathways, including endothelial dysfunction, blood–brain barrier disruption, and increased oxidative stress (Nyúl-Tóth et al., 2024; Elahi et al., 2023; Xiao et al., 2023; Hannawi, 2024). Monocytes, which constitute approximately 20% of peripheral blood mononuclear cells, are central to the immune response and inflammatory processes. In vascular diseases, monocytes migrate into the subendothelial space, where they differentiate into macrophages and engulf oxidized LDL through scavenger receptors and CD36. These macrophages then differentiate into foam cells, releasing pro-inflammatory and pro-oxidant cytokines, which attract additional monocytes and T-lymphocytes, perpetuating vascular inflammation. Monocytes are further influenced by immune stimulants, cytokines, and platelet-derived activation products, contributing to vascular disease progression. Additionally, under inflammatory or prothrombotic conditions, monocytes express tissue factor, adopting a procoagulant phenotype (Ganjali et al., 2018). In contrast, HDL exerts antioxidant, anti-inflammatory, and endothelial-protective effects, influencing coagulation and platelet aggregation (Rhea and Banks, 2021). HDL is recognized as an anti-atherosclerotic factor, inhibiting monocyte-derived tissue factor expression by blocking p38 activation and preventing PI3K inhibition. Moreover, HDL suppresses monocyte activation and regulates the proliferation and differentiation of progenitor cells, thus mitigating inflammatory responses (Ganjali et al., 2018). MHR has emerged as a novel inflammatory marker, reflecting the balance between pro-inflammatory monocytes and anti-inflammatory HDL. Our findings demonstrate that neither monocyte count nor HDL level alone exhibits satisfactory performance in predicting CI or CSVD. However, when combined, these two biomarkers show significantly enhanced predictive capacity for both CI and CSVD. These results further validate the utility of the MHR as a comprehensive indicator integrating pro-inflammatory and anti-inflammatory effects. Studies have demonstrated its association with various chronic inflammation-related vascular diseases (Wu et al., 2024; Ruan et al., 2024; Yang et al., 2025; Xu et al., 2023; Shu et al., 2025), further supporting its role as an indicator of systemic inflammation. Our findings reinforce this concept, revealing a strong correlation between increased MHR levels, CI, and CSVD, further supporting the vital role of chronic inflammation in these conditions. The occurrence of CSVD and CI is driven by a combination of interconnected pathophysiological mechanisms, including chronic hypoperfusion due to atherosclerosis, oxidative stress, BBB disruption, and endothelial dysfunction triggered by inflammatory responses. These mechanisms interact synergistically, collectively promoting the progression of CSVD and its associated cognitive decline (Nyúl-Tóth et al., 2024). First of all, atherosclerosis represents a key pathological process in vascular aging, affecting both large arteries and the microvasculature. Monocytes play a central role in atherosclerosis, contributing to foam cell formation and inflammatory responses. Conversely, HDL mitigates atherosclerosis by inhibiting monocyte activity and exerting anti-inflammatory and antioxidant effects (Ganjali et al., 2018). Clinical and experimental research has established a strong association between peripheral atherosclerosis and CSVD, emphasizing their shared contribution to VCI (Nyúl-Tóth et al., 2024). Although research specifically linking MHR to CSVD and CI is still emerging, numerous studies have demonstrated its association with peripheral atherosclerosis and related diseases. For instance, a study using the Gensini score found that MHR correlated with coronary artery disease severity in a non-linear manner, with a cut-off value of 0.42 (Shu et al., 2025). Additionally, elevated MHR has been related to in-stent restenosis in coronary arteries (Meng H. et al., 2024) and vascular injury-induced carotid plaques (Xi et al., 2022). This research further confirms that MHR and the occurrence of arterial plaque exhibit a non-linear relationship, consistent with the findings of this study (Xi et al., 2022). Furthermore, the well-established relationship between MHR and stroke risk (Xu et al., 2023; Bi et al., 2021; Bolayir et al., 2018), further substantiates its role as a biomarker of ischemic and arteriosclerotic diseases, aligning with our findings. Secondly, endothelial dysfunction is another key contributor to CSVD and VCI. A study on diabetic patients identified MHR as a significant risk factor for endothelial dysfunction, with a 10% increase in MHR correlating to a 35% higher risk of endothelial dysfunction, while a standard deviation increase raised the risk by 61% (Zhang et al., 2024). Several studies have suggested that monocyte-mediated inflammation is a primary driver of endothelial dysfunction (Forbes and Cooper, 2013; Tokarek et al., 2023). Monocytes, derived from bone marrow hematopoietic cells, regulate inflammatory responses, angiogenesis, and vascular remodeling by interacting with endothelial cells (Medrano-Bosch et al., 2023). Activated monocytes, when in contact with damaged or inflamed endothelial cells, trigger the release of pro-inflammatory mediators, ultimately differentiating into macrophages. These macrophages then internalize oxidized LDL, contributing to the formation of foam cells and early-stage atherosclerosis (Savarin et al., 2015; Michalak et al., 2017; Du et al., 2006). In contrast, HDL protects endothelial function by preventing LDL oxidation, reducing endothelial adhesion molecule expression, and inhibiting endothelial apoptosis (Higashi, 2023). Additionally, HDL plays a key role in monocyte regulation, influencing their activation, adhesion, and proliferation into progenitor cells. HDL further enhances eNOS activity, promoting vascular homeostasis and anti-inflammatory effects (Gelaye et al., 2015). Thus, while monocytes exacerbate vascular inflammation and oxidative stress, HDL counteracts these effects, illustrating the pathophysiological relationship between MHR and endothelial dysfunction (Kruit et al., 2004). The vascular endothelium is a fundamental component of the BBB, and its impairment leads to increased permeability, allowing the infiltration of toxic metabolites into periventricular regions, which in turn damages neuronal tissue. Additionally, BBB disruption interferes with the glymphatic clearance of interstitial fluid, further exacerbating neuroinflammation and neuronal injury (Cuadrado-Godia et al., 2018; Nam et al., 2022). BBB dysfunction is a hallmark of CSVD, significantly contributing to demyelination, synaptic impairment, and cognitive decline (Sweeney et al., 2018b; Sweeney et al., 2018a). HDL has been shown to protect the BBB under certain conditions (Rhea and Banks, 2021; Wang Z. et al., 2024). Higher HDL and apolipoprotein A-I levels are associated with reduced BBB damage in multiple sclerosis patients (Fellows et al., 2015). Additionally, higher HDL levels correlate with decreased immune cell infiltration into the central nervous system, further reducing neuroinflammation (Swaminathan et al., 2020). The findings from these clinical studies and mechanistic investigations are consistent with our experimental results, collectively demonstrating the potential of MHR as a promising predictive marker for VCI and CSVD. In fact, there remain several issues that warrant our meticulous consideration. It is well-established that the Apolipoprotein E (APOE) genotype is strongly associated with cognitive impairment and CSVD markers (Lohman et al., 2024). As a key mediator in lipid transport, APOE influences the complex relationship between atherosclerotic processes and neurodegenerative conditions, including dementia. Its three major isoforms differentially regulate lipid homeostasis and neuroinflammatory responses (Nyúl-Tóth et al., 2024). Notably, the APOE4 allele is linked to unfavorable lipid metabolism, exacerbated neuroinflammatory activity, and a higher predisposition to premature cognitive deterioration, white matter lesions, and enlarged perivascular spaces (Nyúl-Tóth et al., 2024; Luo et al., 2017). These effects also elevate the likelihood of developing vascular cognitive impairment and dementia in individuals carrying this allele. Furthermore, APOE4 has been implicated in compromising BBB integrity (Duong et al., 2021). These effects of APOE genotypes are closely linked to the pathological mechanisms underlying CSVD and CI, including inflammation, BBB disruption, and endothelial dysfunction—processes that also intersect with the inflammatory and lipid metabolic pathways represented by the MHR. While our study did not collect APOE genotyping data, we acknowledge that APOE status might modulate the predictive value of MHR, Future studies incorporating APOE genotype and MHR measurements are warranted to clarify their interaction.

This study reveals that MHR is associated with both cognitive impairment and severe CSVD burden. Interestingly, existing research suggests that CSVD and cognitive impairment share common underlying mechanisms. Consistent with previous reports indicating a severe CSVD burden is associated with cognitive impairment, our findings confirm that the severe CSVD burden acts as an independent risk factor for cognitive decline (Li et al., 2021). Moreover, incorporating CSVD burden into multivariable regression models weakened the direct association between MHR and CI, suggesting a mediating role of CSVD burden. Mediation analysis further supports this hypothesis, revealing that severe CSVD accounts for 32.9% of the overall effect. This finding implies that higher MHR levels may contribute to CSVD progression, thereby increasing the risk of CI. Actually, we also conducted additional mediation analyses by reversing the roles of the variables, treating CSVD as the independent variable and MHR as the mediator. The results demonstrated that CSVD likewise mediates the relationship between MHR and cognitive impairment (11.7% of total effect, *p* < 0.001). This suggests a potential bidirectional relationship: (1) Elevated MHR may promote CSVD progression via pro-inflammatory mechanisms; (2) conversely, CSVD-related brain injury could amplify systemic inflammation, further elevating MHR. As with all retrospective analyses, these findings demonstrate association but cannot establish causation. Verification through prospective studies with longitudinal follow-up is essential.

This study offers a novel perspective by simultaneously examining the relationship between MHR and CSVD as well as CI, highlighting the significant mediating role of severe CSVD burden in this association. However, few limitations should be acknowledged. First, as a retrospective study, it cannot establish causal relationships between variables, emphasizing the need for larger prospective studies with greater sample sizes to confirm our findings. Second, monocyte count and high-density lipoprotein levels are dynamic biomarkers, yet this study only measured MHR at admission, failing to capture its temporal variations and their potential impact on CSVD and CI. Additionally, the relatively small sample size limits the generalizability and reliability of the conclusions. Future research should incorporate larger, multi-center cohorts to improve the robustness of the results. Lastly, while cognitive function was assessed using the MMSE, this study did not perform a detailed analysis of specific cognitive sub-domains. Further investigations are warranted to address these limitations and offer a deeper insight into the relationships between MHR, CSVD, and CI.

## 5 Conclusion

In summary, our research highlights significant associations between MHR levels, severe CSVD burden, and CI. Notably, severe CSVD burden plays a key moderating role in the relationships between elevated MHR levels and cognitive decline. Our findings reinforce the idea that higher MHR may facilitate the progression of CSVD, thereby increasing the likelihood of CI.

## Data Availability

The raw data supporting the conclusions of this article will be made available by the authors, without undue reservation.
